# Predatory behavior under monocular and binocular conditions in the semiterrestrial crab *Neohelice granulata*

**DOI:** 10.3389/fnbeh.2023.1186518

**Published:** 2023-05-25

**Authors:** Thomas Harper, Sergio Iván Nemirovsky, Daniel Tomsic, Julieta Sztarker

**Affiliations:** ^1^Instituto de Fisiología, Biología Molecular y Neurociencias (IFIBYNE), Universidad de Buenos Aires, CONICET, Buenos Aires, Argentina; ^2^Instituto de Química Biológica de la Facultad de Ciencias Exactas y Naturales (IQUIBICEN), CONICET, Universidad de Buenos Aires, Buenos Aires, Argentina; ^3^Departamento de Fisiología, Biología Molecular y Celular “Dr. Héctor Maldonado”, Facultad de Ciencias Exactas y Naturales, Universidad de Buenos Aires, Buenos Aires, Argentina

**Keywords:** binocular integration, eye occlusion, crustacean, predatory strategy, stereopsis

## Abstract

**Introduction:**

*Neohelice granulata* crabs live in mudflats where they prey upon smaller crabs. Predatory behavior can be elicited in the laboratory by a dummy moving at ground level in an artificial arena. Previous research found that crabs do not use apparent dummy size nor its retinal speed as a criterion to initiate attacks, relying instead on actual size and distance to the target. To estimate the distance to an object on the ground, *Neohelice* could rely on angular declination below the horizon or, since they are broad-fronted with eye stalks far apart, on stereopsis. Unlike other animals, binocular vision does not widen the visual field of crabs since they already cover 360° monocularly. There exist nonetheless areas of the eye with increased resolution.

**Methods:**

We tested how predatory responses towards the dummy changed when animals’ vision was monocular (one eye occluded by opaque black paint) compared to binocular.

**Results:**

Even though monocular crabs could still perform predatory behaviors, we found a steep reduction in the number of attacks. Predatory performance defined by the probability of completing the attacks and the success rate (the probability of making contact with the dummy once the attack was initiated) was impaired too. Monocular crabs tended to use frontal, ballistic jumps (lunge behavior) less, and the accuracy of those attacks was reduced. Monocular crabs used prey interception (moving toward the dummy while it approached the crab) more frequently, favoring attacks when the dummy was ipsilateral to the viewing eye. Instead, binocular crabs’ responses were balanced in the right and left hemifields. Both groups mainly approached the dummy using the lateral field of view, securing speed of response.

**Conclusion:**

Although two eyes are not strictly necessary for eliciting predatory responses, binocularity is associated with more frequent and precise attacks.

## Introduction

The presence of two image-forming eyes is widespread in the animal kingdom. The benefits of having two eyes (instead of one) include the widening of the visual field and, when there is binocular vision (that is, substantial superposition of the visual fields of both eyes), the improvement of the signal-to-noise ratio of the images, redundancy, seeing around occluders and the possibility of estimating distances by stereopsis (Nityananda and Read, 2017). Crabs’ eyes have thousands of ommatidia distributed around the tip of vertical movable stalks, usually conferring a field of vision of 360 degrees to each single eye (de Astrada et al., 2012). As a consequence, the area of binocular superposition is very wide, covering the whole panorama. There exists extensive binocular integration in brain areas associated with object motion processing and visuo-motor transformation in crabs (Sztarker and Tomsic, 2004; Scarano et al., 2018) but to what extent is binocular information needed to guide specific behaviors remains poorly explored.

*Neohelice granulata* crabs live in mudflats where they are preyed upon by seagulls. While they are commonly described as omnivorous or herbivorous-detritivorous (Bas and Lancia, 2020) they also forage on smaller crabs from the same or other species (Daleo et al., 2003; Tomsic et al., 2017). Their predatory behavior can be readily elicited in the laboratory by a dummy moving at ground level in an artificial arena (Gancedo et al., 2020). Analyzing predatory behavior under diverse conditions, Gancedo and collaborators found that the probability of attacking increases when crabs are close to the tracking line on which the dummy moves and when using small dummies moved at a moderate velocity (Gancedo et al., 2020). Because crabs usually place themselves near the walls, reducing the size of the arena forces animals to be closer to the tracking line and therefore optimizes the chances of eliciting predatory behaviors. Here, we took all this information to set up conditions that maximized the chances of predatory behavior in binocular crabs.

Gancedo and collaborators also determined that crabs use neither apparent dummy size nor its retinal speed as criteria for deciding when to attack. Instead, they start the attack at a specific distance to the dummy (Gancedo et al., 2020). Given that the dummy moves at ground level, estimation of distance to the dummy could be done by using angular declination below the horizon (Ooi et al., 2001; Hemmi and Zeil, 2003). Since *Neohelice* is broad fronted and have well separated eye stalks, stereopsis is theoretically possible too (Zeil and Al-Mutairi, 1996; Layne et al., 1997).

Crabs’ eyes have a band of high vertical resolution around the equator (Zeil and Al-Mutairi, 1996; de Astrada et al., 2012). In *Neohelice granulata* there is also an increased horizontal sampling resolution toward the lateral part of the eye (de Astrada et al., 2012). *Neohelice* crabs can respond to potential predators and preys presented all around, in any azimuthal position (Gancedo et al., 2020). However, when there is need to perform a fast response, for example, when escaping from an approaching predator or pursuing a fleeing prey, they usually turn and run laterally, guaranteeing the fastest velocity of response. During these runs, the image of the potential predator is maintained near the high- resolution lateral pole of one of the eyes (Land and Layne, 1995; Medan et al., 2015). They accomplish this by actively rotating their body since they do not track the image by moving their eyes (Land and Layne, 1995).

In this series of experiments, we set out to explore how occluding one eye affects the predatory behavior of adult crabs. If binocular mechanisms are in use, impaired responses and reduced accuracy of the attacks are expected to occur in monocular crabs.

## Materials and methods

### Animals

Animals were adult male *Neohelice granulata* crabs (Varunidae), 2.7–3.0 cm across the carapace, weighing approximately 17 g, collected in the rías (narrow coastal inlets) of San Clemente del Tuyú, Argentina. Upon arrival at the laboratory, animals were stored in plastic tanks (19 cm × 45 cm × 32 cm, up to 20 individuals per tank) filled to 2 cm depth with artificial sea water (salinity = 10–14 ‰, pH = 7.4–7.6; Coral Pro Salt for Marine Aquarium, manufacturer Red Sea). Animals were kept at 20–26°C, under natural light with water replaced every 48 h. The experiments were run in March-May 2019, between 08:00 and 16:00 h within the first 2 weeks of arrival. Collection was performed in autumn. Animals were not fed during this period.

### Experimental setup and recording procedures

**FIGURE 1:**
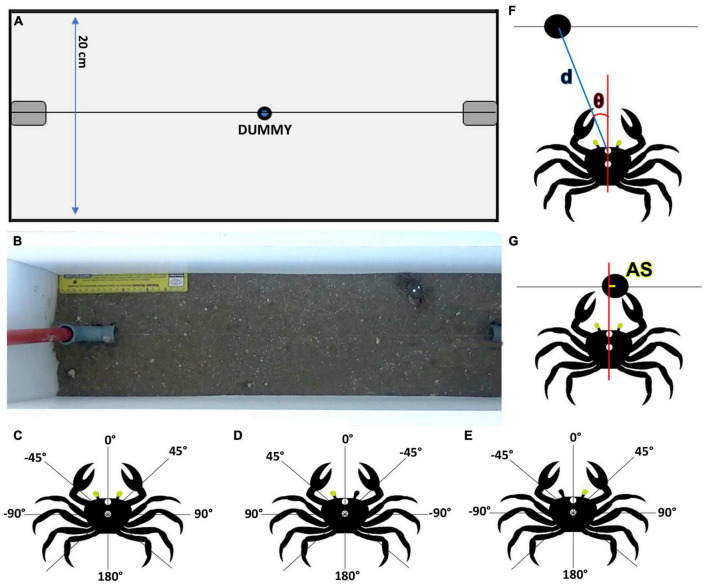
Experimental arena and measurement criteria. Scheme **(A)** and photograph **(B)** of the experimental arena (top view from where the video camera was located). A tracking line located at the center of the arena allowed an attached dummy (1.5 cm diameter) to be pulled, using a manual steering wheel, at ground level between the two sides of the arena. Relative azimuthal position criteria used for the dummy in binocular **(C)** and monocular crabs: left vision **(D)** and right vision **(E)**. Two arcs of 180° were defined. The frontal part (midline between the eyes) was considered 0°. Positive values (0 to 180°) were considered for the right hemifield in binocular crabs **(C)** and for the side of the viewing eye in monocular crabs **(D,E)**. Negative angles (0 to – 180°) were used for the left side for the binocular condition **(C)** and for the side corresponding to the blind eye in monocular conditions **(D,E)**. **(F)** We measured dummy-crab distance (d) and relative position of the dummy (θ) at the beginning of the attack. **(G)** For lunging attacks, the accuracy of the strike (AS) was worked out by projecting a line from the middle of the two outstretched claws and measuring the distance from this line (when it intersected the tracking line) to the middle of the dummy.

Based on previous results we selected experimental conditions that maximally triggered predatory responses (Gancedo et al., 2020). We used a narrow plastic arena (65 cm long × 20 cm wide × 55 cm high) filled to a depth of 5 cm with mud from the crabs’ natural environment (Figures 1A, B). Around all sides of the arena a large curtain prevented uncontrolled visual stimulation. A small black dummy ball (1.5 cm diameter) was attached to a thin fishing line that allowed its movement at a speed of approximately 200 mm/s and at ground level. The tracking line was situated in the center of the arena, 10 cm from the longer edges and passed through vertical plastic pipes located over the shorter edges (Figures 1A, B). The tubes held the dummy out of sight and connected the line to a series of pulleys which allowed an operator to wind it. Suspended above the center of the arena, was a video camera (Sony HDR-CX440) remotely operated by a smartphone with the app Imaging Edge Mobile 7.2.1. To help the tracking of the crabs, two spots of white correction fluid (liquid paper) were added to the carapace (one between the two eyestalks and another in the middle of the carapace, Figures 1B–E). Each crab was tested five times in alternating directions, with an intertrial interval (ITI) of 3 min to curtail habituation. Animals were left undisturbed in the arena for 10 min before the trials began.

### Eye occlusion

A total of 66 crabs were used: 22 control (binocular crabs, Figure 1C), 22 that had the right eye occluded (left vision, Figure 1D) and 22 that had the left eye occluded (right vision, Figure 1E). Occlusion was achieved by using opaque black spray paint (Kuwait, Aerofarma, Argentina) applied with a fine brush. To this end, each crab was held in a clamp by its carapace and the eye to be painted was hold in position by placing a wire support under the eye. After the paint had dried the support was removed and crabs were placed individually in glass containers filled with 2 cm of saltwater. The containers were stored in racks in a quiet room with controlled temperature and natural light where crabs were allowed to recover for 24 h. To test the blinding method, four animals were completely blinded by painting both eyes. During 20 trials there was no response to the dummy.

### Response criteria and measurements

As in a previous study by Gancedo et al. (2020) behavioral responses were characterized into four categories: freezing response (FR), when the animal stopped walking and remained still for the rest of the trial; no response (NR), when the crab did not change its behavior (remained still during the whole trial or kept moving without acknowledging the dummy); avoidance response (AR), when the animal moved away from the dummy and predatory response (PR) if there was movement toward the dummy. Among PR, responses were separated in complete attacks (CA) if the crab approached and tried to catch the dummy and incomplete attacks (IA) if the animal started to approach but interrupted the action. Videos containing CA were analyzed using the tracking software Tracker 5.1.2 and further separated into successful attacks (SA, if the crab touched or caught the dummy) and unsuccessful attacks (UA; if it missed and did not touch the dummy). Trials in which the dummy had already gone into the tube when the crab attacked it were not considered. We calculated the azimuthal angular position of the dummy in relation to the crab when the decision of attack was taken considering two arcs of 180 degrees, with 0°corresponding to the midline between the eyes at the front of the crab. Positive values (0 to 180°) were considered for the right hemifield in binocular crabs and for the side ipsilateral to the viewing eye in monocular crabs (Figures 1C–E). Negative angles (0 to −180°) were used for the left side for the binocular condition and for the side corresponding to the blind eye in monocular conditions (Figures 1C–E). The final orientation of the crab relative to the dummy was determined at the moment any part of the animal first touched the tracking line or got to the closest position to the dummy. It was measured as the angle between the crab’s midline and the dummy’s center with the crab’s frontal marker spot as the vertex. We also measured the distance between the crab’s frontal marker spot and the center dummy at the start of the attack (d, Figure 1F).

Predatory response strategies were defined considering the movement of the dummy in relation to the crab: if the dummy was approaching the crab, they were defined as interception attacks; if it was moving away, as pursuits. Lunge behavior involved the crab jumping forward very quickly and reaching out to grab the dummy with both claws. For lunges, the accuracy of the strike (AS) was worked out by projecting a line from the middle of the two outstretched claws. Distance from this line (when it intersected the tracking line) to the middle of the dummy was measured (Figure 1G). If the value was 0 this meant the crab was precise and caught the dummy exactly between the two claws.

### Statistical analysis

Statistical analyses were performed in R (R Core Team, 2022). Multinomial regression models generated through the *nnet* package (Venables and Ripley, 2002) were used to identify changes in responses, with Likelihood Ratio Tests (LRT) between full models and nested incomplete models used to probe the factors (condition, trial, individuals) and possible interactions. Response-rate estimates and *post hoc* comparisons were carried out with the *emmeans* package using Tukey’s method (Lenth, 2022). Mixed effects logistic regression models were evaluated with the *lme4* package (Bates et al., 2015) to assess if individuals contributed random intercepts to the models. Whenever the 0 was located within the random effects’ 95% confidence intervals, it was considered null and taken off the model (confirmed by LRT or the model being singular with random σ^2^≈0), recurring to a fixed effects logistic regression instead. Likelihood ratio tests were used to assess the different factors. Linear mixed effects models were carried out with the *lme4* package as before; whenever necessary, data was transformed by its logarithm to achieve normality. Again, effects were tested by LRTs and marginal estimates and *post hoc* comparisons by use of the *emmeans* package. When random effects were negligible (as described above), simple linear models were fitted instead. Data presented in the text represent means ± SEM unless indicated otherwise.

## Results

### Changes in dummy-induced behaviors under binocular and monocular vision

**FIGURE 2:**
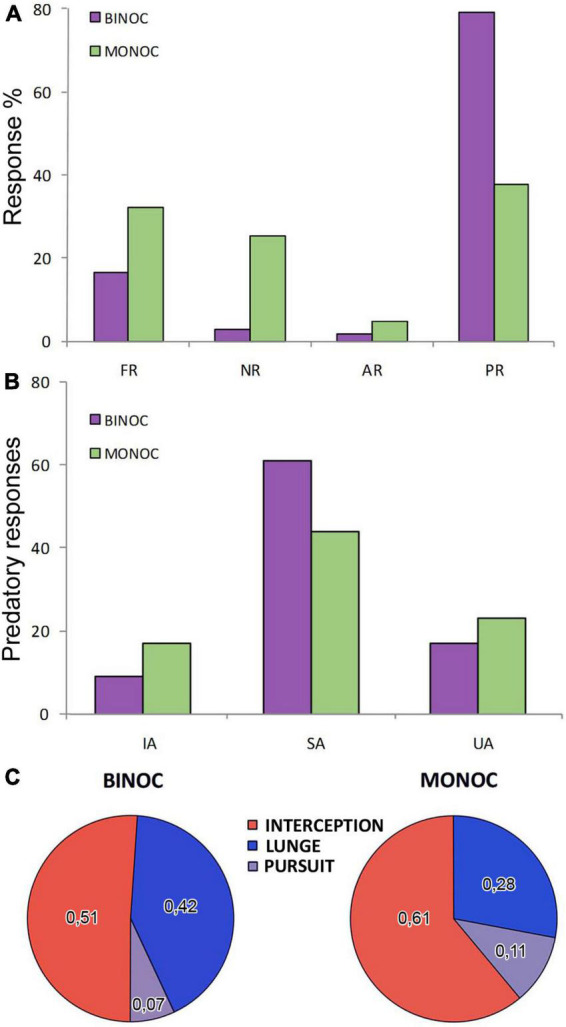
Response differences in binocular and monocular crabs. **(A)** Percentage of responses evoked by the dummy in crabs (purple: binocular; green: monocular) categorized into four mutually exclusive responses: freezing response (FR; if crabs were moving and stopped upon the movement of the dummy), no response (NR; if no change in behavior was evoked by the dummy), avoidance response (AR; if crabs moved away from the dummy) and predatory response (PR; if crabs moved toward the dummy). **(B)** Observed predatory responses were split into incomplete attacks (IA) and, for complete attacks: successful attacks (SA; if the crab caught or touched the dummy) and unsuccessful attacks (UA; if it did not). **(C)** Proportion of the 3 attack strategies used for the complete predatory responses. Interception represents an attack triggered while the dummy was approaching the crab (red). Lunge behavior, if the crab jumped forward very quickly (blue) and pursuit if the crab attacked while the dummy was moving away (gray).

We tested the responses of 66 adult male crabs (22 control binocular crabs, 22 with only left eye vision and 22 with only right eye vision) to the motion of a small dummy (Figures 1A–E) with size and speed (see section “Materials and methods”) on the range of those displayed by the small crabs *Neohelice* preys upon. Each animal received 5 trials (ITI: 3 min) producing a total of 110 trials in each condition. The observed behaviors were separated in four categories (Figure 2A). Freezing responses (FR) were considered when the animal stopped walking and remained still for the rest of the trial. No responses (NR) when the crab did not change its behavior (remained still during the whole trial or kept moving without acknowledging the dummy). Avoidance responses (AR) when the animal moved away from the dummy and predatory responses (PR) when there were movements toward the dummy.

Contrasting with what occurred in larger experimental arenas (Gancedo et al., 2020; Salido et al., 2023) in the narrow arena used here, binocular crabs showed mainly PR (in almost 80% of the trials, Figure 2A), with a minority of the animals displaying FR and almost none having NR or AR. Monocular crabs produced a very different distribution of behaviors [Figure 2A, LRT: χ^2^_(*df*=3)_ = 59.9, *p* = 6.11 × 10^–13^]. They could still perform predatory behavior, although there was an acute descent on its probability, attacking in less than 40% of the trials (model estimated probabilities: MONOC = 0.382 ± 0.0325 vs. BINOC = 0.791 ± 0.0386; *p* = 4.71 × 10^–06^). This was accompanied by augmented probabilities of FR (almost doubling the one seen with binocular responses; MONOC = 0.323 ± 0.031 vs. BINOC = 0.164 ± 0.0351; *p* = 0.0512) and NR (about ten times more common than in the binocular group; MONOC = 0.245 ± 0.0279 vs. BINOC = 0.0273 ± 0.0155; *p* = 4.75 × 10^–05^).

### Predatory responses

Crabs display different forms of predatory responses. They can run toward the dummy while the target is approaching (interception attack, Supplementary Movie 1), they can wait until the dummy is near and jump forward very quickly (lunge behavior, Supplementary Movie 2) or they can attack while the dummy is moving away from the crab and perform a pursuit behavior (Supplementary Movie 3). After starting the predatory behavior, they can abandon the attack (incomplete attack, IA) or they can complete the attack by trying to catch the dummy (complete attack, CA). CA can be further subdivided in successful attacks (SA) if the crab touches or catches the dummy with the claws or legs, or unsuccessful attack (UA) if it misses.

Considering this, we explored if, in addition to attacking less, monocular crabs showed changes in the parameters of the response, the strategies used or the efficacy in the predatory responses. SA were the most common outcome both in binocular and monocular crabs (Figure 2B). Nonetheless, monocular crabs did show a tendency to be less effective in their attacks than binocular crabs reducing the number of SA and increasing the number of IA and UA (Figure 2B). In fact, they showed less commitment to complete the attacks [logistic model estimates for the probabilities of completing the attack SA + UA: MONOC = 0.798 ± 0.0438 vs. BINOC 0.897 ± 0.0326; LRT: χ^2^_(df=1)_ = 3.28, *p* = 0.0699] and a reduced probability of success in the complete attacks [MONOC = 0.657 ± 0.058 vs. BINOC 0.782 ± 0.047; LRT: χ^2^_(df=1)_ = 2.83, *p* = 0.0924]. The lack of more pronounced differences in these parameters may be related with other changes observed in the behavior of monocular crabs. Accordingly, when analyzing the strategies used in complete attacks, we observed differences between binocular and monocular crabs (Figure 2C). In particular, a diminished proportion of lunging behavior was noticeable in monocular crabs. A logistic regression showed that monocular crabs chose to lunge fewer times (proportional to the other types of attack), producing this behavior in about one fourth of the times (0.279 ± 0.0574) compared to binocular crabs that displayed this behavior almost half of the times (0.425 ± 0.0579). The two conditions were very close to be differentiated (OR: 0.523 with 95% CI: 0.249, 1.07).

Monocular crabs performed a high proportion of interception behavior (the crab attacking the dummy before it reaches the point closest to the animal; Figure 2C). Pursuit behavior (in which the crab following the dummy while it is moving away from the animal; Figure 2C) was infrequent in both groups mostly due to the narrow size of the arena, where crabs cannot be far enough of the tracking line as to display long distance chases.

**FIGURE 3:**
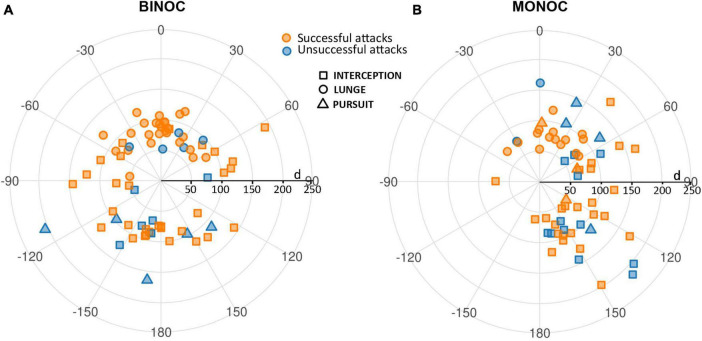
Relative azimuthal position of the dummy respective to the frontal midline of the crab at the start of the attack for all complete attacks in binocular **(A)** and monocular **(B)** crabs. The midline between the eyes is set at 0°, positive values represent the right hemifield in binocular crabs and the side ipsilateral to the viewing eye in monocular crabs, negative angles represent the left side and the side corresponding to the blind eye. Relative azimuthal position is plotted against dummy-crab distance. The strategies used are represented by the different symbols: squares for interception, circles for lunge and triangles for pursuit attacks. The outcome of each attack is denoted by the color: orange for successful and blue for unsuccessful attacks.

Figure 3 displays relative azimuthal position information (dummy-crab) at the start of the predatory behavior for all complete attacks in binocular and monocular crabs. It includes information regarding the strategy used (interception, lunge, pursuit) and the outcome of the attack (successful or unsuccessful). The angular position and distance of successful and unsuccessful attacks were similar and therefore were not separated in further analyses. The different predatory strategies showed a clustered distribution and will be analyzed separately in the following sections.

**FIGURE 4:**
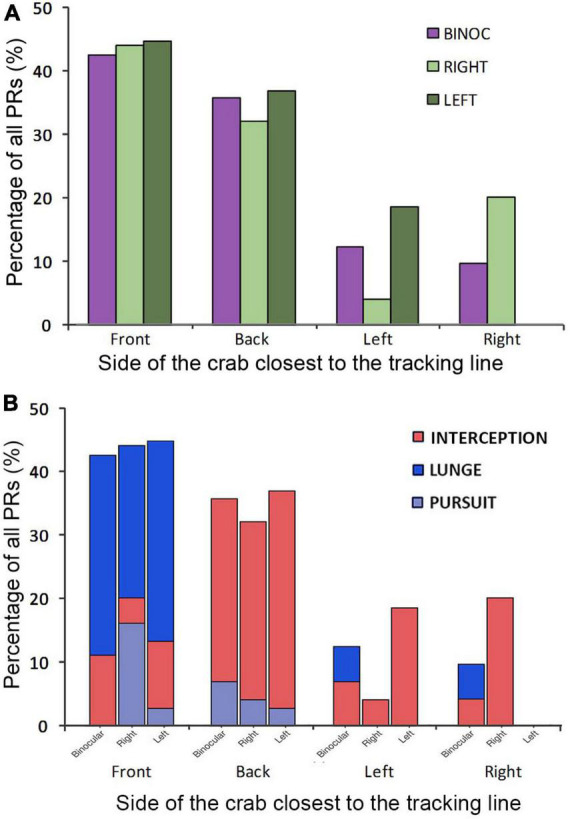
**(A)** Orientation of the crabs in the arena at the beginning of the complete attack trials (when the dummy first began to move). The *x*-axis categories display the side of the crab (Front, Back, Left, and Right) closest to the tracking line. Purple bars: binocular crabs; Green bars: monocular crabs split in right vision (light green) and left vision (dark green). **(B)** Attack strategies used depended on the orientation of the crabs in the arena at the beginning of the trial. Percentage of use of the different strategies of predatory responses (PRs; Interception: red; Lunge: blue; Pursuit: gray) in relation to the side of the crab (Front, Back, Left, and Right) closest to the tracking line and the viewing condition: binocular, right and left vision.

Overall, in coincidence with previous results (Gancedo et al., 2020), binocular crabs showed a homogeneous distribution of PR all around the animal (Figure 3A). In contrast, monocular crabs showed very few attacks when the dummy was on the side of the blinded eye (negative angles; Figure 3B). This side bias is only moderately explained by the orientation of the crabs at the beginning of the trials. As can be seen in Figure 4A both monocular (split in left and right vision crabs in this analysis) and binocular crabs showed a similar augmented predisposition to be frontal or backward respect to the tracking line when the dummy began to move (they usually locate near the longer walls of the arena). In these two initial positions a side biased is not predicted. Conversely, when monocular crabs were located sideways with respect to the tracking line they were mainly oriented with the viewing eye closer to the tracking line (notice in Figure 4A that all animals with left vision that were sideways, placed themselves with the left eye closer to the tracking line while the majority of the right vision crabs had the right eye closer to it). Monocular crabs that had the viewing eye closest to the dummy at the start of the trial, attacked before it passed the crab’s midline (90%, with 79% SA) while the few ones that had the blind eye closer to the dummy waited until it was closest to the viewing eye to attack (87%, with a 55% SA).

In Figure 4B, we further separated the data to include the predatory strategy used depending on the initial orientation of the crab. The more evident observation is that the animals’ initial position greatly influences the strategy used. Crabs facing the tracking line lunged frequently, while those backward or lateral to the tracking line used mainly interception behavior. In monocular crabs, no lunges were seen in the lateral positions (Figure 4B).

Regarding the dummy-crab distance at the start of the attack, there were no differences between binocular (86.2 ± 4.28 mm) and monocular crabs (88.3 ± 4.46 mm; LRT: χ^2^_*df = 1*_ = 0.119, *p* = 0.730).

We decided to further compare the way monocular and binocular crabs attacked by analyzing the two main strategies used in this arena: interception and lunge attacks.

### Interception attacks

**FIGURE 5:**
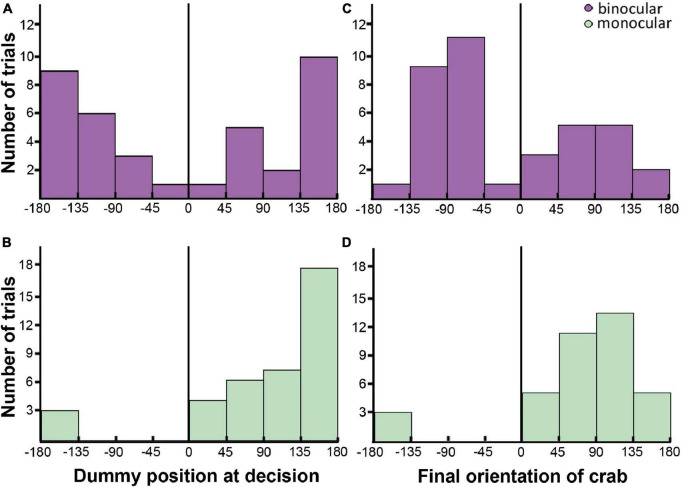
Parameters of interception attacks in binocular [**(A,C)**: purple] and monocular [**(B,D)**: green] crabs. **(A,B)** Bar plots showing the number of trials where the crabs had the dummy positioned in the different relative azimuthal positions (Bin size 45°; | 0–45°| = front; | 45–135°| = lateral; | 180–135°| = back) when the decision to attack was expected to be made (170 ms before the first approach movement). **(C,D)** Bar plots showing the number of trials where the crabs oriented at different angles respective to the dummy at the end of the attack (Bin size 45°; | 0–45°| = front; | 45–135°| = lateral; | 180–135°| = back). In **(B,D)** a marked side bias in monocular crabs toward the seeing eye side is noticeable. The black line marks the frontal midline that separate the two hemifields.

The decision to initiate PR was estimated to happen 10 frames (170 ms) before the first dummy-directed approaching movement, to include the time delay that exists between the decision to move and the motion of the animal (Oliva and Tomsic, 2012). The relative azimuthal position of the dummy at decision-making time was differently distributed in both groups (Figures 5A, B; negative values represent the blind eye side in monocular crabs, irrespective of whether it was the right or the left one, and left side in binocular animals). The distribution of the bars shows that both binocular and monocular crabs had the dummy preferentially positioned in latero-backward regions (|45–180°—) when deciding to use interception (Figures 5A, B). This is consistent with the data shown in Figure 4B. The difference is that, while attacks in binocular crabs were triggered with the dummy located either on the left or the right side, monocular attacks were mainly triggered in the uncovered eye side (positive values). Using a logistic regression, we confirmed that monocular crabs were biased against the occluded eye side, with an estimated probability of 0.92 ± 0.045 of the interceptions occurring on the seeing eye side (95% Confidence Interval: 0.777–0.974; 0.5 is not included in the interval meaning there is a side bias), contrasted to the binocular crabs that showed no preference (0.49 ± 0.082; 95% CI: 0.332–0.644; 0.5 is included in the interval meaning there is no bias). The difference between the two groups was corroborated by LRT: χ${}_{d\,f=1}^{2}$ = 18, *p* = 2.2 × 10^–05^.

The distance at the start of the attack was not significantly different between binocular and monocular crabs (binocular: 91.1 ± 4.97 mm; monocular: 93.3 ± 4.86 mm; LRT: χ${}_{d\,f=1}^{2}$ = 0.12; *p* = 0.729).

Lastly, we evaluated the final orientation of the crab when it reached the dummy (the angle of the dummy relative to the frontal midline of the crab at the moment of reaching the tracking line; Figures 5C, D). We found a significant effect of the eyesight condition on this parameter (LRT: χ${}_{d\,f=1}^{2}$ = 23.9; *p* = 1 × 10^–06^), with an unbiased distribution in binocular crabs (estimated probabilities being close to 0.5: 0.405 ± 0.0807; 95% CI: 0.261–0.568) but biased toward the uncovered eye in monocular crabs (estimated probabilities: 0.919 ± 0.0449; 95% CI: 0.777–0.974). Most animals oriented in a lateral way (|45–135°|), which guarantees visual acuity and a faster attack with the difference that binocular animals favored both lateral directions while monocular animals only moved toward the uncovered eye (Figures 5C, D).

### Lunge attacks

**FIGURE 6:**
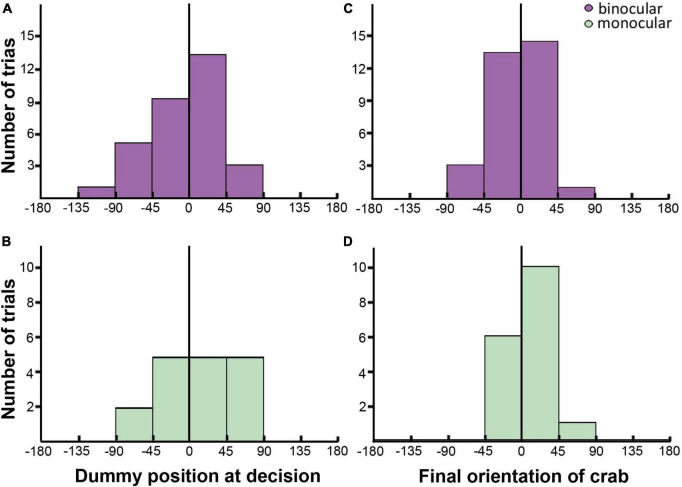
Parameters of lunge attacks in binocular [**(A,C)**: purple] and monocular [**(B,D)**: green] crabs. **(A,B)** Bar plots showing the number of trials where the crabs had the dummy positioned in different relative azimuthal positions (Bin size 45°; | 0–45°| = front; | 45–135°| = lateral; | 180–135°| = back) when the decision to attack was expected to be made. **(C,D)** Bar plots showing the number of trials where the crabs oriented at different angles respect to the dummy at the end of the attack (Bin size 45°; | 0–45°| = front; | 45–135°| = lateral; | 180–135°| = back). The distribution of binocular and monocular data is very similar. The black line marks the frontal midline that separate the two hemifields.

Even though monocular crabs lunge less, the overall characteristics of lunge attacks were similar in monocular and binocular crabs. A cursory inspection of monocular parameters associated with lunge (Figures 6B, D) shows a similar distribution between the side of the blind eye (negative values) and the side of the uncovered eye (positive values). Indeed, the relative angular position of the dummy at lunge decision-making was similar in monocular and binocular crabs (Figures 6A, B; LRT: χ${}_{d\,f=1}^{2}$ = 0.23; *p* = 0.63), lacking the bias seen in interception attacks and showing no side preference in binocular (0.516 ± 0.0898; 95% CI: 0.345–0.683) nor monocular crabs (0.588 ± 0.1190; 95% CI: 0.352–0.790). There was neither a difference between monocular and binocular crabs nor a side preference in the final attack orientation (Figures 6C, D; LRT: χ${}_{d\,f=1}^{2}$ = 1.19; *p* = 0.275; binocular: 0.484 ± 0.0898, 95% CI: 0.317–0.655; monocular crabs: 0.647 ± 0.1160, 95% CI: 0.404–0.832). The attack distance was also similar in the two conditions (binocular: 81.6 ± 4.61 mm; monocular: 83.6 ± 5.03 mm; LRT: χ${}_{d\,f=1}^{2}$ = 0.865; *p* = 0.352). Nonetheless, when looking at the accuracy of the attacks (how centered the attack was, 0 indicating the crab caught the dummy exactly between the two claws, Figure 1G), differences between the groups were noticeable. Binocular animals were more precise in their attack compared to monocular crabs [BINOC = −4.49 ± 1.61 mm vs. MONOC = −10.40 ± 2.13 mm; *F*_(1_,_44)_ = 5.04, *p* = 0.0299].

## Discussion

In this report we described the changes produced in the predatory behavior of *Neohelice granulata* mud crabs, evoked by the movement of a small dummy at ground level, when the vision of one of their eyes was occluded. In these crabs, that possess periscope-like eyes with a 360° visual span each, occluding one eye does not restrict the visual field of the animals, although it reduces information content. We observed a deep reduction in the probability of predation in monocular crabs accompanied by a rise in the probability of not responding or actively freezing. Additionally, several characteristics of the predatory responses changed as discussed in the following sections.

### Although two eyes are not strictly necessary, binocular vision warranties more numerous and accurate predatory responses

A first important outcome of the present study is that monocular crabs could still perform predatory behaviors and therefore two eyes are not strictly necessary for eliciting attacks. Nonetheless, several results indicate an impaired performance. The clearest is the stark reduction in the probability of initiating an attack (from about 80% in binocular crabs to less than 40% in monocular crabs). In addition, monocular crabs were less committed to complete the attacks, showed a reduction in the probability of success and varied their use of predation strategies. Therefore, restricting the vision to only one eye clearly changes the way crabs predate. Binocular vision can be used to derive information about depth by comparing information from both eyes, to increase contrast sensitivity, to improve the ability to see around obstacles and to detect objects in cluttered environments (Read, 2021). The impairments seen in the predatory responses of monocular crabs could be associated to a diminished visual input (only half the information is reaching the brain) or to the impossibility of imaging the same spot with both eyes (use of binocular vision). Results from ongoing experiments painting the right (or left) halves of both eyes so as to limit the amount of information without precluding binocular vision show that the number of attacks made by these crabs is higher than the amount made by monocular crabs, supporting the need of binocularity for optimal predatory responses (Kalesnik Vissio and Sztarker, unpublished data).

### Different factors including monocular vision affect which predatory strategies are favored

Crabs can use different strategies when attacking a potential prey. They can intercept the moving target when it is approaching, they can wait until the dummy has reached the nearest distance and lunge with a quick frontal jump or they can wait until the dummy is retreating and pursue it. Present results support the idea that the chosen strategy depends on diverse factors including the size of the arena, the position/orientation of the crabs with respect to the dummy and if they can see the stimulus with one or both eyes. These factors are actually related to each other. As seen in Figure 4, in the small arena used in the present study, animals were frequently located frontally or backward to the tracking line and favored lunge and interception behaviors, respectively. Pursuits were infrequent in this arena probably because crabs need more freedom of movement and larger distances to develop such behavior. We observed only 7% of pursuits in binocular crabs compared to 34% reported in a larger arena (45 cm wide; Gancedo et al., 2020).

Other factors such as hunger can also modify predatory frequency and used strategies. Salido et al. (2023) noticed an important difference in the probability of attacking between fed and unfed crabs, the latter attacking more. However, such difference disappeared if only crabs that were very close to the tracking line were considered. This probably involves an economic decision based on the effort required for attacking vs. the need for food. If the possible prey is very close the attack will be triggered since it is energetically cheap. Instead, if the crab is far away from the tracking line, a long chase of the dummy will be costly and therefore avoided unless feeding is required. These results are highly relevant to the present findings. The low costs involved in attacking the dummy when the crab is positioned very close to the tracking line might drive predatory responses even in suboptimal conditions (e.g., under monocular vision).

In bigger arenas, where crabs are further away from the tracking line, the predatory response is elicited much less frequently (36.5% in Gancedo et al., 2020 vs. 79% in binocular crabs in the present results). Gancedo et al. (2020) did not report lunge attacks. They observed a high frequency of interception attacks (66%). In the present experimental conditions binocular (51%) and, even more often, monocular crabs (61%) also favored interception attacks, running toward the dummy while it was approaching the crab.

### Monocular crabs showed a side bias when using interception attacks and were less accurate during lunge attacks

When performing interception attacks, monocular crabs detected, turned and approached the dummy favoring the side of the viewing eye while binocular crabs did not present a side bias. In accordance with Figure 4B, data from Figures 5A, B suggest that this behavior is frequently chosen when crabs see the dummy in the backward or lateral positions. While the dummy elicited attacks in both hemifields in binocular crabs, monocular crabs responded primarily toward the side of the viewing eye, meaning the attack was seldom triggered when the dummy was seen by the medial region of the seeing eye. After the decision of attacking was taken, animals from both groups turned and approached the dummy using the lateral field of view (Figures 5C, D), securing visual acuity and speed of response.

As seen in Figure 4B crabs that were initially positioned frontally to the tracking line were more prone to use lunge attacks. Present results show that crabs reduced the use of lunging when only monocular cues were available. The fact that the accuracy of the attack (how centered the attack was) was significantly reduced in monocular crabs supports an impairment in the execution of this behavior when only one eye is available. Other parameters of the lunge attacks, however, were similar in monocular and binocular conditions, with no side bias. Crabs decided to attack and approached the dummy when positioned in a centered frontal region (Figure 6).

### Monocular performance affects other behaviors in *Neohelice granulata*

We have previously explored how occluding one eye affects other behaviors in this crab species (Sztarker and Tomsic, 2008; Barnatan et al., 2019). Considering escape responses, monocularly deprived crabs also displayed significantly weaker responses (about half in intensity) than animals with binocular vision (Sztarker and Tomsic, 2008). In these experiments, as in the present series, occlusion of the eye was performed 24 h before testing. The timing was chosen to reduce the stress component induced by the experimental manipulations associated with occluding the eye. Previous experiments confirmed that this is an appropriate interval since if the paint is removed on the second day, animals recover normal escape responses (Sztarker, 2000; Sztarker and Tomsic, 2008).

We also explored monocular performance during optomotor responses (Barnatan et al., 2019). Here, results were more complex. Monocular crabs actually displayed stronger optomotor responses than binocular crabs but only when the ipsilateral field of the viewing eye experimented progressive (front-to-back) motion stimulation. If regressive motion stimulation was used, monocular responses were practically abolished (Barnatan et al., 2019).

The different behavioral outcomes are surely related with the way the underlying neural networks are organized to transmit, add or subtract the information originating from the two eyes. We know that binocular integration starts at the level of the lobula of *Neohelice* (Sztarker and Tomsic, 2004). In fact, we have described different types of binocular neurons showing diverse computation of ipsilateral, contralateral and binocular inputs. Some neurons responded with similar strength to monocular and binocular input, some showed stronger responses to binocular input and others weaker responses. Some received comparable inputs from both eyes while others received stronger ipsilateral or contralateral inputs (Scarano et al., 2018). Additional experiments are needed to further explore and understand the circuitry behind each behavior.

### Is there evidence for stereopsis in the predatory behavior of *Neohelice*?

The observation that the distance of attack did not change between monocular and binocular groups in any of the evaluated predatory responses might indicate that the distance was estimated monocularly by angular declination. More likely, however, a precise distance calculation may not be needed to initiate predation of the dummy in the narrow enclosure used in the present experiments. Both in binocular and monocular groups, attack distances were much smaller (∼8–9 cm) than the mean value reported for larger arenas (about 15 cm; Gancedo et al., 2020; Salido et al., 2023) where a fixed crab-dummy distance was reported in predatory behaviors. If we had used a wider arena, measured distances may have corresponded to earlier stages of the predatory response and differences in the distances between monocular and binocular conditions might have been evidenced. Naturally, the percentage of predatory behaviors would have been greatly reduced and we would have been left with too few monocular attacks as to make a good characterization of the responses.

Establishing the use of stereopsis is challenging. Stereopsis has been suggested to be possible in several invertebrates including dragonflies (Olberg et al., 2005), damselflies (Schröder et al., 2018), beetles (Bauer, 1981), robber flies (Wardill et al., 2017), and crabs (Scarano et al., 2018; Gancedo et al., 2020) among others. However, so far, only two invertebrates have been conclusively added to the list of animals able to estimate distance by stereopsis, the praying mantis (Maldonado and Rodriguez, 1972; Rossel, 1983; Nityananda et al., 2016) and the cuttlefish (Feord et al., 2020). In both cases, the ultimate proof has been achieved by modifying the visual perception of the animal with anaglyph 3D images and color filter lenses while measuring the distance of the ballistic attacks produced. In praying mantis, neurons proposed to be involved in the neural network mediating stereopsis have been found (Rosner et al., 2017). Neurons with similar properties have been described in damselflies (Supple et al., 2020) and in *Neohelice granulata* crabs (Scarano et al., 2018) providing strong candidates for animals that use stereopsis. The range of depth estimation is limited by the interocular distance, which is quite small in most insects (Olberg et al., 2005) but considerably broader in the case of *Neohelice granulata*. Theoretical calculations [based on Burkhardt et al., 1973; E∞ = (b/2)/tan (Δφ/2)] taking into account a typical interocular distance in an adult crab (*b* = 2.2 cm) and the interommatidial angles in the frontal visual region (average: 0.7 based on de Astrada et al., 2012) render that this crab would be able to estimate distances up to 180 cm.

Monocular praying mantis greatly reduce the number of attacks compared to binocular animals (Maldonado et al., 1967). Similarly, present results indicate that for taking the decision to initiate a predatory behavior the availability of both eyes is extremely important in crabs. The presence of binocular cues also improved the proportion of complete and successful attacks. Yet, a definite proof of the use of stereopsis in crabs is still pending. To do this, the study of ballistic predatory responses triggered at specific distances by virtual stimuli would be ideal, as it was done for mantises and sepias (Nityananda et al., 2016; Feord et al., 2020). The predatory response of *Neohelice* triggered by a small dummy moving on the ground seems to be suitable for such a purpose.

## Data availability statement

The raw data supporting the conclusions of this article will be made available by the authors, without undue reservation.

## Author contributions

JS and DT helped in study concept and design and obtained funding. TH helped in acquisition of data. SN helped in the statistical analysis. TH, SN, and JS helped in analysis, figures design, and interpretation of data. JS helped in drafting of the article and study supervision. TH, SN, DT, and JS helped in critical revision of the manuscript. All authors had full access to all the data in the study and take responsibility for the integrity of the data and the accuracy of the data analysis.

## Supplementary material

Supplementary Movie 1 Example of a successful interception attack in a monocular crab (right vision). The video is shown in slow motion (half normal speed) to improve visualization of the attack.

Supplementary Movie 2 Example of a successful lunge attack in a binocular crab. The video is shown in slow motion (half normal speed) to improve visualization of the attack.

Supplementary Movie 3 Example of a successful pursuit attack in a monocular crab (right vision). The video is shown in slow motion (half normal speed) to improve visualization of the attack.
